# The Effect of Severe Coronary Calcification on Diagnostic Performance of Computed Tomography-Derived Fractional Flow Reserve Analyses in People with Coronary Artery Disease

**DOI:** 10.3390/diagnostics14161738

**Published:** 2024-08-10

**Authors:** Iva Žuža, Tin Nadarević, Tomislav Jakljević, Nina Bartolović, Slavica Kovačić

**Affiliations:** 1 Department of Diagnostic and Interventional Radiology, Clinical Hospital Centre Rijeka, 51000 Rijeka, Croatia; tin.nadarevic@gmail.com (T.N.); nina.bartolovic@gmail.com (N.B.); slavica.kovacic@yahoo.com (S.K.); 2 Faculty of Medicine, University of Rijeka, 51000 Rijeka, Croatia; tomislav.jakljevic@medri.uniri.hr; 3 Clinic for Heart and Vessel Diseases, Clinical Hospital Centre Rijeka, 51000 Rijeka, Croatia

**Keywords:** computed tomography, coronary angiography, fractional flow reserve, sensitivity and specificity

## Abstract

Background: Negative CCTA can effectively exclude significant CAD, eliminating the need for further noninvasive or invasive testing. However, in the presence of severe CAD, the accuracy declines, thus necessitating additional testing. The aim of our study was to evaluate the diagnostic performance of noninvasive cFFR derived from CCTA, compared to ICA in detecting hemodynamically significant stenoses in participants with high CAC scores (>400). Methods: This study included 37 participants suspected of having CAD who underwent CCTA and ICA. CAC was calculated and cFFR analyses were performed using an on-site machine learning-based algorithm. Diagnostic accuracy parameters of CCTA and cFFR were calculated on a per-vessel level. Results: The median total CAC score was 870, with an IQR of 642–1370. Regarding CCTA, sensitivity and specificity for RCA were 60% and 67% with an AUC of 0.639; a LAD of 87% and 50% with an AUC of 0.688; an LCX of 33% and 90% with an AUC of 0.617, respectively. Regarding cFFR, sensitivity and specificity for RCA were 60% and 61% with an AUC of 0.606; a LAD of 75% and 54% with an AUC of 0.647; an LCX of 50% and 77% with an AUC of 0.647. No significant differences between AUCs of coronary CTA and cFFR for each vessel were found. Conclusions: Our results showed poor diagnostic accuracy of CCTA and cFFR in determining significant ischemia-related lesions in participants with high CAC scores when compared to ICA. Based on our results and study limitations we cannot exclude cFFR as a method for determining significant stenoses in people with high CAC. A key issue is accurate and detailed lumen segmentation based on good-quality CCTA images.

## 1. Introduction

Coronary CT angiography (CCTA) is a well-established, noninvasive imaging test for assessing coronary artery disease (CAD). Its high negative predictive value enables the effective exclusion of significant CAD, reducing the need for further imaging tests or catheter-based coronary angiography [1,2,3,4]. However, in the presence of coronary artery calcification, the specificity of CCTA in assessing stenosis severity is reduced [5,6,7,8]. Calcified coronary plaques can lead to an overestimation of stenosis severity on CCTA, primarily due to blooming and beam-hardening artifacts, which obscure the luminal border. This misclassification may influence the treatment decision making among people with suspected CAD and future risks [8]. Furthermore, CCTA is an anatomical test that does not provide physiological information about the hemodynamic significance of a certain stenosis. This limitation is particularly challenging in people with morphologically intermediate stenoses, as it becomes difficult to determine if certain stenosis is causing ischemia [9]. According to the European Society of Cardiology (ESC) 2019 guidelines for the diagnosis and management of people with chronic coronary syndrome, if the pretest probability of obstructive CAD is high and CCTA results are uncertain or non-diagnostic regarding functional significance, individuals should be referred for additional functional testing for ischemia (Class I recommendation). This further testing can be either non-invasive or invasive [2]. Negative testing for ischemia supports a shift towards medication management, reducing the number of people referred to the catheterization lab for invasive procedures. A class IIa recommendation includes invasive fractional flow reserve (iFFR) measurements to assess the functional significance of intermediate coronary lesions and to guide subsequent treatment decisions [2]. However, clinical implementation of iFFR may be limited, mostly because of its invasive nature, the duration of the procedure, and reimbursement issues. 

Computed tomography-derived fractional flow reserve testing (CT-FFR) was developed to provide both morphological and hemodynamic assessment of coronary lesions using a single noninvasive test. CT-FFR was approved for clinical use by the US Food and Drug Administration (FDA) in 2014. This method includes three-dimensional (3D) modeling of the entire coronary tree to calculate FFR values from CCTA and is available as a web-based service provided by HeartFlow, Inc. (Redwood City, CA, USA) [10]. A simplified, one-dimensional (1D) FFR analysis (cFFR) has been developed by Siemens Healthcare (Forchheim, Germany). This method calculates FFR using the cross-sectional area of the vessels, making broad assumptions about how blood flows through arteries. It has been proposed as a method that could be performed at on-site workstations; however, until now, it has only been available for research purposes [11,12].

Irregular heart rates and highly calcified lesions most severely impact image quality and present the greatest challenges for image interpretation [13]. Image quality similarly affects the diagnostic performance of cFFR, primarily by influencing coronary artery segmentation, which is a crucial step in creating the anatomical coronary model. Previous studies have demonstrated the effectiveness of CT-FFR in individuals with CAC scores above 400 [8,14]. Evidence on the performance of 1D cFFR analysis in calcified vessels is limited [12,14,15,16].

The aim of our study was to evaluate the diagnostic performance of noninvasive cFFR derived from CCTA, compared to the ICA as a reference standard, in detecting hemodynamically significant stenoses in people with high calcium scores (>400). We hypothesize that cFFR will provide additional information and improve specificity compared to CCTA interpretation alone, thereby offering valuable insights for further clinical management. 

## 2. Materials and Methods

### 2.1. Participants

This research was performed in accordance with the Declaration of Helsinki principles. Approval from the institutional review boards was given, and all participants provided written informed consent. We conducted a single-center retrospective study in which we included consecutive participants aged ≥18 years who underwent CCTA with clinical suspicion of CAD. Clinical reasons for ordering CCTA included suspicion of obstructive coronary atherosclerotic plaques, suspicion of ischemic heart disease and patients with stable symptoms where noninvasive stress tests were inconclusive of coronary artery disease [17]. Participants who underwent the CT exam from May 2021 to March 2023 and had a CAC score of more than 400 were included in the study. All participants subsequently underwent ICA as a reference standard. Exclusion criteria were renal dysfunction (glomerular filtration rate < 30 mL/kg/1.73 m^2^), allergy to iodine contrast media, previous coronary intervention or coronary bypass surgery, suspected acute coronary syndrome, myocardial infarction within 30 days before or between CCTA and the invasive procedure, severe artifacts caused by arrhythmia that prevented the assessment of coronary artery stenoses or unequivocal chronic total occlusions (CTO), and absence of ICA. Participant data were retrieved from the electronic medical records and included demographic data (age and gender), comorbidities (diabetes, arterial hypertension, dyslipidemia), smoking status, prior myocardial infarction and clinical follow-up (choice of optimal medical therapy, percutaneous coronary interventions and/or coronary artery bypass grafting). Optimal medical therapy refers to the prescription of single antiplatelet or novel oral anticoagulant therapy, intensive hypolipidemic therapy and antihypertensive agents according to the European Society of Cardiology guidelines for the diagnosis and management of patients with chronic coronary syndrome [2]. In the final group of included participants, there was no missing data or loss to follow-up.

### 2.2. CT Data Acquisition

All examinations were performed on a Dual Source CT scanner (SOMATOM Flash, Siemens Healthineers, Forchheim, Germany). Prior to scanning, sublingual nitroglycerin was administered to each participant, and depending on the heart rate, intravenous application of beta blockers was given in order to achieve a heart rate of nearly 60 beats/min. First, a prospective electrocardiogram-triggered scan acquired at 55–65% of the RR interval with 3 mm slice thickness was used as a non-enhanced acquisition in order to assess the CAC score. Secondly, a contrast-enhanced electrocardiograph (ECG) gated scan was performed, and based on the participant’s heart rate, prospective triggering (adaptive-sequential) or retrospective gating was used. Contrast-enhanced CT images were reconstructed using iterative reconstruction with the following parameters: kernel, medium smooth convolution kernel (I30f); slice thickness, 0.75 mm; increment, 0.5 mm; field-of-view, 200 × 200 mm.

### 2.3. CCTA Evaluation

CCTA exams were evaluated by a board-certified radiologist with 6 years of experience in cardiovascular radiology (T.N.) on a dedicated workstation with commercially available software (Syngo.via VB60A_HF08 Siemens Healthineers). The assessments included CAC score calculation, measurement of morphological degree of stenoses expressed in percentages relative to normal vessel lumen and categorizing degree of stenoses according to the Coronary Artery Disease Reporting and Data System (CAD-RADS) [18]. Coronary artery stenosis severity was categorized as minimal (<25%), mild (25–49%), moderate (50–69%), and severe (70–99%) by visual assessment. For the purposes of this study, coronary artery stenoses were grouped and categorized into non-significant (<50%) and significant (≥50%). The same reader assessed the quality of acquired images, categorizing them into interpretable and uninterpretable. The reader was blinded to the clinical information, cFFR and ICA results as well as clinical outcomes. 

### 2.4. cFFR Analysis

cFFR analyses were performed using an on-site machine learning-based algorithm (cFFR, version 3.1.0; Siemens Healthineers) [19]. The analysis was performed by a board-certified radiologist with 10 years of experience in cardiovascular radiology (I.Z.) on previously acquired CT coronarography exams, using part of the cardiac cycle which was assessed to have the best image quality and least artifacts. Utilizing previously acquired CCTA exams, the part of the cardiac cycle with the best image quality and the fewest artifacts was selected for assessment. The preprocessing to generate the model of the coronary artery tree was semiautomatic. The system generated centerlines and luminal contours or coronary arteries which were manually modified and optimized by the reader. Once all centerlines and volumes were corrected, manual marking of coronary artery stenoses was performed. Following the marking of stenoses, the algorithm computed cFFR values in all locations of the coronary arteries and reconstructed the color-coded coronary artery tree. cFFR values considered for the purposes of this study were the ones calculated 2 cm distal to the morphologically most severe stenosis of the right coronary artery (RCA), left anterior descending artery (LAD) and left circumflex artery (LCX) [20,21]. A cFFR value of 0.80 was considered to be the cut-off to differentiate positive (<0.80) and negative lesions (≥0.80). The reader was blinded to the results of the CCTA and ICA as well as clinical outcomes. Measurements were conducted on each coronary artery, and a per-vessel analysis was performed.

### 2.5. Invasive Coronary Angiography

ICA exams and stenosis measurements were performed by an interventional cardiologist with 15 years of experience in coronary interventions (T.J.) using a clinically approved interventional angiography system (Artis Q, Siemens, Siemens Healthineers AG, Forcheim, Germany). Using left or right radial artery access, left and right coronary arteries were visualized with corresponding diagnostic catheter and iodine contrast agent. At least two orthogonal projections were filmed for each coronary artery. All atherosclerotic lesions were analyzed quantitatively with the QCA assessment program (CAAS–RUBO, Pie Medical Imaging, Maastricht, The Netherlands). The degree of stenosis was expressed as a percentage relative to the adjacent normal vessel lumen. The interpreter of the ICA results was aware of the CCTA findings, as CCTA was part of the clinical workup conducted prior to ICA. However, the interpreter of ICA was blinded to the results of cFFR.

### 2.6. Statistical Analysis

Statistical analysis was performed using Statistica for Windows, version 14.0.0.15. (Statsoft, Inc., Tulsa, OK, USA). The normality of the distribution of quantitative data was tested using Kolmogorov–Smirnov test. Separate analyses were made for each coronary artery (RCA, LAD and LCX). Non-normally distributed and continuous data are presented with median and interquartile range (IQR) while ordinal and categorical variables are presented as numbers and percentages. Comparison of non-parametric continuous data was performed by a Mann–Whitney U test. To determine the diagnostic accuracy of cFFR, receiver operating characteristics analysis was used to calculate the AUC and sensitivity and specificity, and Youden’s index was used to calculate the optimal threshold for cFFR positivity. All statistical results were considered significant at the value of *p* < 0.05.

## 3. Results

### 3.1. Baseline Characteristics

**Figure 1:**
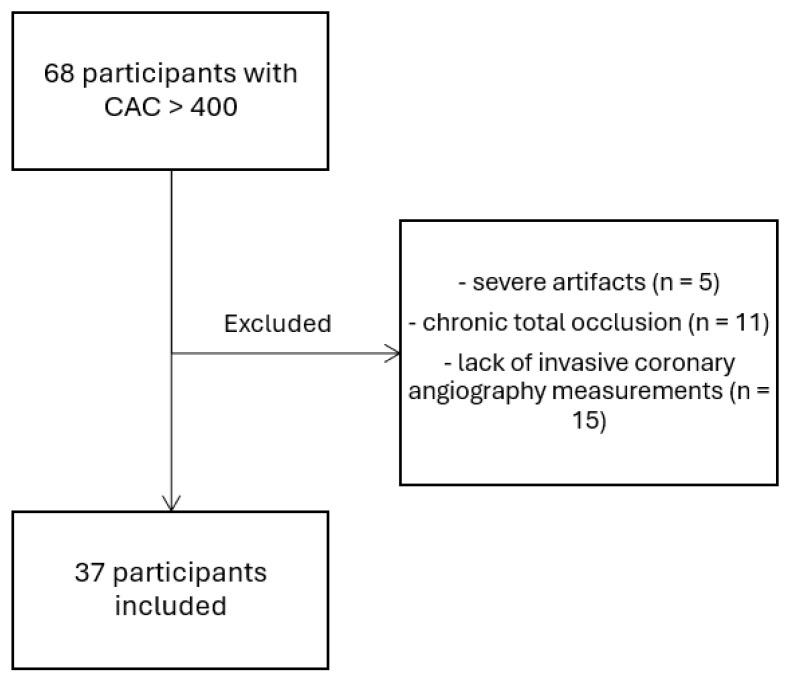
Flowchart of selected participants.

From May 2021 to March 2023, we included 68 participants who underwent elective CCTA with clinical suspicion for coronary artery disease and were found to have a CAC score of more than 400. Out of 68 participants, 5 were excluded due to severe artifacts, 11 due to chronic total occlusion of any coronary artery, and 15 were excluded due to lack of invasive coronary angiography measurements (Figure 1). Finally, our study included 37 participants who met all the inclusion criteria. Table 1 shows participant characteristics. 

### 3.2. CAC Score

All participants showed high values of CAC score (median 870, IQR 642–1370), with a minimum value of 417 and a maximum of 3299, respectively. In order to determine differences in the distribution of CAC scores, all participants were divided into groups with cFFR values of <0.8 and ≥0.8 and ICA results indicating stenoses < 50% and ≥50%. Grouping was performed according to each coronary artery. In cases of different cFFR groups, there were no significant differences in all three coronary arteries: RCA (median 183, IQR 45–475 versus median 248, IQR 61–560: *p* = 0.59), LAD (median 308, IQR 189–572 versus median 425, IQR 258–650: *p* = 0.35), and LCX (median 121, IQR 17–201 versus median 205, IQR 81–241: *p* = 0.18). Distribution of calcium in participants with <50% stenosis on ICA compared to ones with ≥50% stenoses showed no significant differences in all three coronary arteries: RCA (median 180, IQR 41–488 versus median 350, IQR 213–861: *p* = 0.18), LAD (median 354, IQR 234–586 versus median 439, IQR 177–860: *p* = 0.72), and LCX (median 129, IQR 17–231 versus median 168, IQR 33–499: *p* = 0.42).

### 3.3. CCTA and cFFR Diagnostic Accuracy

Diagnostic accuracies of CCTA were calculated on a per-vessel analysis principle. Using ICA as a reference standard, the AUC for RCA was 0.639 with a calculated sensitivity of 60% and a specificity of 67%. The AUC for LAD was 0.688 with a sensitivity of 87% and specificity of 50%, while the AUC for LCX was 0.617 with a sensitivity of 33% and a specificity of 90%, respectively. All CCTA imaging data were deemed adequate and appropriate for further cFFR analyses. 

**Figure 2:**
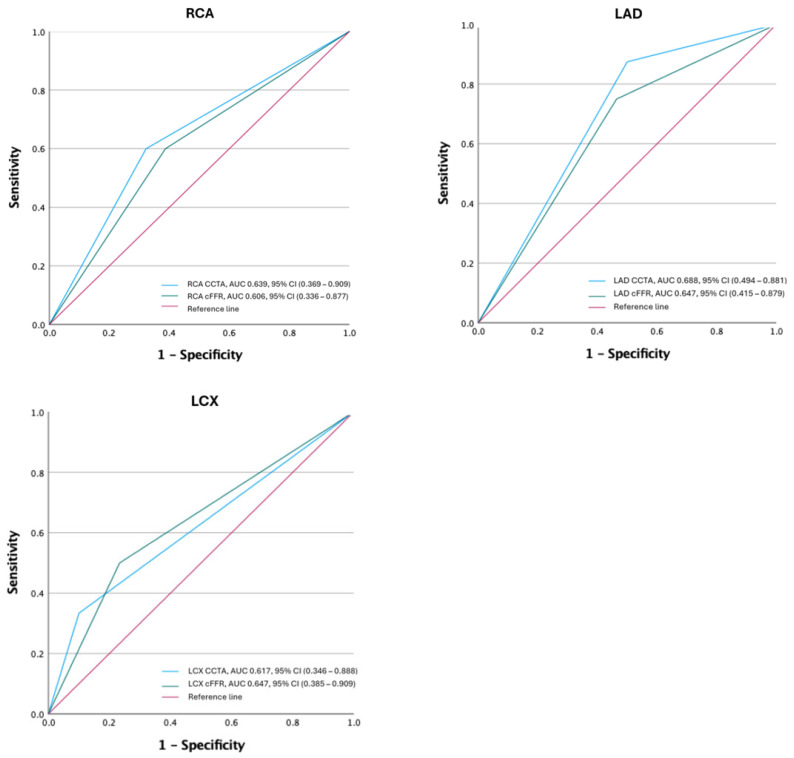
ROC curves of CCTA and cFFR for each coronary artery. RCA—right coronary artery, LAD—left anterior descending artery, LCX—left circumflex artery, AUC—area under the curve, CCTA—coronary computed tomography angiography, cFFR—one dimensional (1D) FFR analysis.

**Figure 3:**
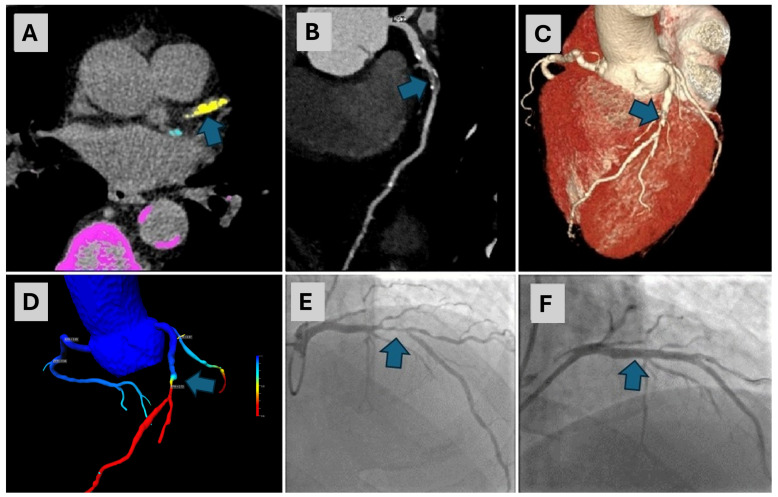
An 80-year-old male with a CAC score of 868.5, exhibiting prominent calcifications in the LAD artery ((**A**)—arrow), underwent CCTA. The CCTA revealed severe stenosis in the mid-segment of the LAD artery ((**B**)—arrow, (**C**)—arrow). A cFFR test confirmed a significant stenosis in the same segment ((**D**)—arrow), with a cFFR value of less than 0.7. Consequently, ICA was performed, which confirmed 80% stenosis in the LAD ((**E**)—arrow). This was followed by percutaneous coronary intervention (PCI) with stenting and the final angiography exam showed complete revascularization ((**F**)—arrow). CAC—coronary artery calcium, LAD—left anterior descending, CCTA—coronary computed tomographic angiography, cFFR—computed fractional flow reserve, ICA—invasive coronary angiography, PCI—percutaneous coronary intervention.

**Figure 4:**
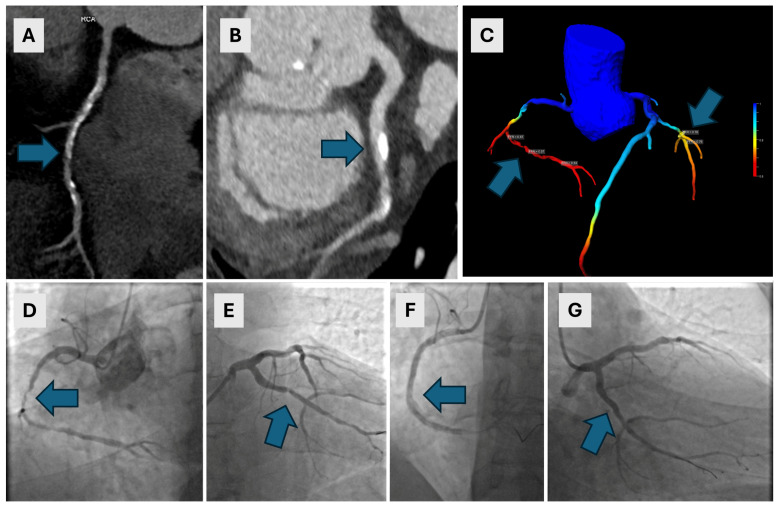
A 63-year-old male with a history of arterial hypertension and nonspecific arrhythmia underwent CCTA. The CAC score was 1406.7. Extensive calcified and mixed plaques were found in the RCA, with severe stenosis of up to 85% and in the RCA ((**A**)—arrow), and moderate stenosis of 65% in the LCX ((**B**)—arrow). A cFFR confirmed the significance of both lesions, with cFFR values of less than 0.7 in the RCA and 0.76 in the LCX ((**C**)—arrows). ICA revealed long 80–99% stenoses in the middle segment of the RCA ((**D**)—arrow) and a 90% stenosis in the proximal LCX ((**E**)—arrow). Immediate PCI of the RCA was performed with complete revascularization ((**F**)—arrow) which was complicated by anaphylactic shock. A few days later, after premedication, PCI of the LCX was performed with stent implantation and complete revascularization ((**G**)—arrow). CCTA—coronary computed tomographic angiography, CAC—coronary artery calcium, RCA—right coronary artery, LCX—left circumflex artery, cFFR—computed fractional flow reserve, ICA—invasive coronary angiography, PCI—percutaneous coronary intervention.

**Figure 5:**
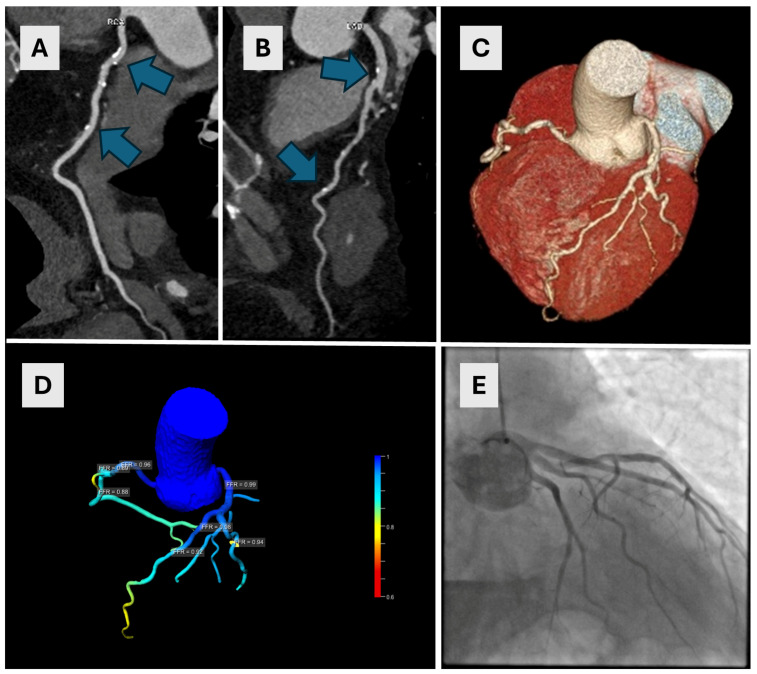
A 54-year-old male underwent CCTA for suspected ischemic heart disease. The CAC score was 417. Calcified plaques were found in both the RCA ((**A**)—arrows) and LAD ((**B**)—arrows) causing moderate stenoses (50–52% in the LAD and 63% in the proximal RCA). Image (**C**) shows a VRT reconstruction image of coronary arteries with multiple small plaques on LAD. A cFFR test showed no significant stenoses (**D**) which was subsequently confirmed on ICA (**E**). CCTA—coronary computed tomographic angiography, CAC—coronary artery calcium, RCA—right coronary artery, LAD—left anterior descending artery, VRT—volume rendering technique, cFFR—computed fractional flow reserve, ICA—invasive coronary angiography.

All CCTA exams were used to calculate cFFR values for each coronary artery. Diagnostic accuracy parameters for cFFR were as follows: the AUC for RCA was 0.606 with a calculated sensitivity of 60% and a specificity of 61%; the AUC for LAD was 0.647 with a calculated sensitivity of 75%, and a specificity of 54%. Likewise, the AUC for LCX was 0.647 with a sensitivity of 50% and a specificity of 77%, respectively. Using Youden’s index to assess the cFFR cut-off value, which provides the best sensitivity and specificity, the cut-off value was 0.7. Using this value, diagnostic accuracy values for RCA, LAD and LCX were the following: an RCA sensitivity of 60%, a specificity of 71%; a LAD sensitivity of 63%, a specificity of 61%; and an LCX sensitivity of 66% and a specificity of 76%, respectively. There were no significant differences between AUCs of CT coronary angiography and cFFR for each vessel (all *p* > 0.05). Relevant diagnostic accuracy values are listed in Table 2, and ROC curves for RCA, LAD and LCX are presented in Figure 2. Representative images of three participants are presented in Figure 3, Figure 4 and Figure 5.

## 4. Discussion

In this study, we evaluated the diagnostic performance of cFFR as an added value to the standard CCTA in individuals with a high calcium score (greater than 400), using ICA as the reference standard. Our results did not show any significant benefit in using cFFR in determining ischemia-specific lesions, and the AUCs of CCTA and cFFR were lower than expected in the literature. The results of this study are at odds with other similar studies that provided information on the diagnostic accuracy of cFFR in participants with high CAC scores where the AUCs were at least 0.71 [8,22,23,24,25,26]. Our results did not show the benefit of cFFR in determining significant hemodynamically significant stenoses. 

Mickley et al. performed a prospective multicenter study that included participants with a CAC of >399. In total, 260 participants were included, all of whom underwent CCTA, cFFR and ICA with/without iFFR measurements as the reference standard. Per-vessel analysis using colocation cFFR measurements showed sensitivity and specificity of 82% and 75% with an AUC of 0.81 [22]. Nørgaard et al. examined the influence of coronary calcification on the diagnostic performance of cFFR and included 214 participants, of which 53 participants had 83 vessels with a CAC of more than 415. All participants underwent CCTA, cFFR and iFFR measurements as the reference standard. Per-vessel analysis showed a sensitivity of 82%, a specificity of 46%, and an AUC of 0.91 [8]. Tang et al. performed a retrospective multicenter study that included 338 participants who underwent CCTA, cFFR and iFFR measurements. Per-vessel analysis which included 27 participants with a CAC of ≥400 yielded sensitivity and specificity of 91% and 100% with an AUC of 0.97 [23]. Tao et al. examined the effect of CAC on the diagnostic accuracy of cFFR in a multicenter retrospective study. The authors included 128 participants who underwent CTA, cFFR, and iFFR measurements. In total, 41 participants had CAC ≥400 and the analysis reported per-vessel sensitivity 100%, specificity 95% with an AUC of 0.98 [24]. Tesche et al. also examined the influence of CAC on the diagnostic performance of cFFR. They included participants who underwent CCTA, cFFR and iFFR measurements. A total of 68 vessels showed a CAC score of ≥400, and the analysis yielded cFFR sensitivity and specificity of 91% and 68% and with an AUC of 0.71 [25]. Zhao et al. reported the diagnostic performance of cFRR across different CAC groups. They included 305 participants with 348 target vessels in a prospective multicenter clinical trial. All participants underwent CCTA, cFFR and iFFR measurements. The analysis included 25 participants with a CAC of ≥400 and yielded a sensitivity and specificity of 93% and 100% with an AUC of 0.98 [26]. Ma et al. performed a systematic review for assessing the diagnostic accuracy of cFFR and CCTA at different levels of CAC score. One of the per-vessel analyses for the accuracy of cFFR included four primary studies with a total of 306 participants with CAC scores of ≥400 and yielded a summary sensitivity and specificity of 86% and 63%, with an AUC of 0.72 [27]

CAC often imposes a challenge for the interpretation and determination of stenoses. On CCTA exams due to partial volume effects and blooming artifacts, it is often difficult to accurately contour the lumen in the stenosis which can impact the measurements and results [28]. The same issue is for the cFFR calculations. CAC and blooming artifacts can impose a challenge for accurate lumen calculations. Another issue is adequate image quality which impacts the results of the cFFR analysis. Precise cFFR measurements rely on accurate anatomical models based on good-quality images [29]. In our study, all CT studies were assessed as interpretable with adequate image quality. 

CCTA and invasive coronary angiography are important modalities in detecting coronary artery disease, but they are not the sole tools used for diagnosis and management. In the context of chronic coronary syndrome, a multimodality approach is necessary to choose the best treatment option for each patient. Noninvasive diagnostic modalities employed in the diagnostic workup of patients with chronic coronary syndrome include echocardiography, CCTA, cardiac magnetic resonance (CMR), positron emission tomography (PET) and single photon emission computed tomography (SPECT). Based on the risk stratification of each patient, the integration of these imaging modalities allows for a comprehensive evaluation of chronic coronary syndrome and provides information on coronary anatomy, myocardial perfusion and function. Each modality has its strengths and limitations, and the choice of imaging depends on the specific clinical scenario, patient characteristics, and availability of resources. A personalized approach, utilizing the strengths of each modality, is crucial for accurate diagnosis and effective management of chronic coronary syndrome [30].

In recent years, new CT advancements have emerged which show promising potential for improving diagnostic accuracy and image quality. Such technologies are Photon-Counting Detector (PCD) CT and spectral CT. PCD CT has emerged as groundbreaking CT technology, enabling multi-energy scanning by directly converting X-ray photon energies into electrical signals. The advantages of this new CT system include improved spatial resolution, reduced blooming artifacts and the ability to separate calcium from images using material decomposition algorithms, which may allow for a more accurate estimation of luminal stenosis [31,32]. Studies using PCD CT showed a reduction in stenosis overestimation, improved image resolution and significant changes in CAD-RADS categorization/clinical recommendations [33,34]. Spectral CT technology uses two layers of detectors to simultaneously collect low- and high-energy data, consequently being able to generate conventional poly-energetic and dedicated spectral images [35]. Studies showed the benefit of this technology in improving contrast enhancement in suboptimal post-contrast CT studies, reduction in artifacts, and improvement of coronary artery lumen definition [36,37,38]. These technical advancements show promising results in significantly improving the accuracy of CT findings.

Our study included 37 participants with a CAC score of more than 400. The number of included participants is low, and our study may be underpowered to produce higher accuracy estimates than expected. However, despite our small sample size, other diagnostic studies included a similar number of participants (N < 70) and reported a higher AUC for cFFR in participants with a severe CAC [8,24,25]. 

To assess the accuracy of cFFR, other studies used iFFR measurements as the reference standard for diagnostic accuracy assessment, whereas we used ICA. iFFR provides physiological information about the severity of stenosis; however, it can be complex, not readily available, and costly to perform. Consequently, it is not widely used as invasive coronary angiography, which serves as the basis for many clinical decisions. Invasive angiography offers detailed information regarding the location and extent of stenotic lesions, which is crucial for planning interventions, particularly in complex cases. Most clinical guidelines and protocols still rely on invasive coronary angiography for diagnosing coronary artery disease and planning revascularization procedures [2,39]. We sought to assess the performance of cFFR compared to the current standard of practice in our institution. A limitation of using this reference standard is the reliance on subjective intraprocedural measurements and/or visual nonquantitative assessments of potential ischemia-related lesions, which may affect the results. Additionally, an important limitation in our data is the uneven distribution of participants with significant (≥50% stenosis) and non-significant stenosis on invasive coronary angiography; 5 participants were found to have significant stenosis, while 31 had non-significant stenosis. This imbalance may have also impacted our results. However, our results provide preliminary insight into challenges posed by severe calcification which warrants further investigations in a larger number of patients.

Several methods for cFFR measurements are reported in the literature: immediately distal to the stenotic lesion (i.e., non-stenotic adjacent vessel lumen), the lowest cFFR value in the entire coronary artery, 1–2 cm distal to the stenotic lesion and ΔcFFR (i.e., ratio of cFFR value proximal and distal to the stenosis). Due to the highest reported accuracy and recommendations, we performed measurements 1–2 cm distal to the stenosis [21]. An issue that may have impacted the results is a single-reader assessment and difficulties in determining the cFFR value in the exact location of 1–2 cm distal to the stenosis. The same single-reader limitation is also valid for the CCTA morphological stenosis interpretation. A different single reader was assessing CCTA, cFFR and ICA findings, so we could not assess inter-reader variability and the potential impact of measurements on our data. 

The use of per-patient and per-vessel analysis should always be guided by the scientific question we want to answer with our research. In this study, we chose to specifically target people with severe CAC scores, and we wanted to see the impact of high calcium scores on cFFR diagnostic performance. We performed per-vessel analysis because of the diagnostic importance of detecting ischemia-specific lesions. Another limitation of this study is the inclusion of participants with a total CAC of ≥400, and not participants with a CAC of each coronary artery being ≥400. This might have impacted the results, and such analysis might have provided more robust results, but this criterion would have excluded even more participants from the study. In our analysis and the results reported, we did not show the benefit of cFFR, i.e., the diagnostic performance was not better than CCTA alone. 

Our study has several limitations which may have impacted the results. Apart from the retrospective study innate flaws, small sample size, invasive coronary angiography alone without iFFR measurements, uneven distribution of positive and negative reference standard results, and potential errors in measurements may have impacted the results and thus being in odd with all other clinical studies.

## 5. Conclusions

In this study, we aimed to assess the diagnostic accuracy of cFFR and compare it with CCTA in identifying hemodynamically and morphologically significant lesions in individuals with high CAC scores. The diagnostic accuracy of cFFR and CCTA was found to be poor in individuals with high CAC scores when compared to invasive coronary angiography, rather than iFFR. Our findings indicate that both methods exhibit limited diagnostic accuracy in this population. However, it is important to note that our study’s limitations, including a small sample size and lower accuracy values compared to larger studies, preclude us from definitively concluding that cFFR is an unreliable method for detecting significant stenoses in individuals with high CAC scores. Crucially, to ensure reliable cFFR accuracy, meticulous and precise lumen segmentation based on high-quality CCTA images is essential.

## Figures and Tables

**Table 1:** Participant demographics.

Feature	Participants (All 37)
age (years)	67; 9
female, *n* (%)	15 (41)
diabetes, *n* (%)	13 (35)
arterial hypertension, *n* (%)	35 (95)
dyslipidemia, *n* (%)	35 (95)
smoking, *n* (%)	15 (41)
prior myocardial infarction, *n* (%)	0 (0%)
CAC score	870; 642–1370
Follow-up:	
optimal medical treatment *n* (%)	20 (54)
percutaneous coronary intervention *n* (%)	14 (38)
coronary artery bypass grafting *n* (%)	3 (8)

Age value is given as mean; standard deviation. CAC score is given in median; interquartile range.

**Table 2:** Diagnostic accuracy data on cFFR and CCTA for diagnosing ischemia-specific lesions.

	**CCTA**			
	**AUC**	**95% CI**	**Sensitivity**	**Specificity**
RCA	0.639	0.369–0.909	60%	67%
LAD	0.688	0.494–0.881	87%	50%
LCX	0.617	0.346–0.888	33%	90%
	**cFFR**			
	**AUC**	**95% CI**	**Sensitivity**	**Specificity**
RCA	0.606	0.336–0.877	60%	61%
LAD	0.647	0.415–0.879	75%	54%
LCX	0.647	0.385–0.909	50%	77%

## Data Availability

The original contributions presented in the study are included in the article, further inquiries can be directed to the corresponding author.
